# Hospitalisation Resulting from Medicine-Related Problems in Adult Patients with Cardiovascular Diseases and Diabetes in the United Kingdom and Saudi Arabia

**DOI:** 10.3390/ijerph13050479

**Published:** 2016-05-09

**Authors:** Abdullah Al Hamid, Zoe Aslanpour, Hisham Aljadhey, Maisoon Ghaleb

**Affiliations:** 1 Department of Pharmacy, Pharmacology and Postgraduate Medicine, School of Life and Medical Sciences, University of Hertfordshire, Hatfield AL10 9AB, UK; z.aslanpour@herts.ac.uk (Z.A.); m.ghaleb@herts.ac.uk (M.G.); 2 Medication Safety Research Chair, College of Pharmacy, King Saud University, Riyadh P.O. Box 2457, Saudi Arabia; haljadhey@ksu.edu.sa

**Keywords:** cardiovascular diseases, diabetes, prevalence, medicine-related problems, hospitalisation, treatment effectiveness, adverse drug reactions, polypharmacy

## Abstract

Cardiovascular diseases (CVDs) and diabetes (DM) are two interrelated conditions that have a heavy morbidity and mortality burden worldwide. Patients with the two conditions usually take multiple medicines and thus are more susceptible to medicine-related problems (MRPs). MRPs can occur at any stage of the treatment process and in many cases can lead to unplanned hospitalisations. The aim of the study was to determine the prevalence of hospitalisation resulting from MRPs in adult patients with CVDs and/or DM and to identify the main causes, risk factors, and medicine classes involved. A retrospective study included 300 adult patients from two hospitals, one in the United Kingdom and one in Saudi Arabia. To identify MRPs, medical records were reviewed for demographic data, clinical data, laboratory assay, and prescription records. A total of 197 (65.7%) patients had MRPs, of which less than 10% were severe. The main problems were lack of treatment effectiveness and adverse drug reactions. Moreover, polypharmacy and patient non-adherence were the main risk factors contributing to MRPs. The main medicine classes associated with MRPs were insulin and antihypertensive medicines. Further research should address the pharmaceutical care processes employed in treating CVDs and DM, and to empower patients/healthcare providers in tackling MRPs.

## 1. Introduction

According to the World Health Organisation, by 2020 chronic conditions, such as cardiovascular diseases (CVDs) and diabetes (DM), will be the major source of morbidity worldwide [1]. Currently, CVDs account for 48% of deaths worldwide, whereas DM accounts for 3% [2]. In the UK, CVDs are responsible for 191,000 deaths per year [2], and more than 2.5 million people are diagnosed with DM [2]. The situation is even more complex in Saudi Arabia (SA), where CVDs and DM account for 35% and 7% of all deaths, respectively [3].

A medicine-related problem (MRP) is defined as “an event or circumstance involving drug therapy that actually or potentially interferes with their desired outcome” [4]. MRPs can occur at any stage of the medication process. Patients with CVDs and DM take multiple medicines from diverse pharmacological classes, raising the likelihood that MRPs may occur.

More specifically, hospitalisation resulting from MRPs is a major concern to both patients and healthcare professionals due to its tremendous health and economic burdens. Many meta-analyses and studies have revealed that medicine-related hospitalisations (MRH) are responsible for 2.4%–6.2% (2.4%–3.6% in Australia and 3.6%–6.2% in North America) of the total number of medical admissions [5,6,7]. Markedly, the main classes of medicines that account for MRH include medicines prescribed for CVDs and DM [8,9,10,11,12]. Moreover, the major risk factors contributing to MRPs are either related to the patient (*i.e.*, physiological and life-style) and/or to the medicines [13]. However, to the best of our knowledge no study to date has highlighted the particular influence of these risk factors on CVDs, and only one study investigated the association between these risk factors and MRPs in diabetic patients [14].

The UK has a well-established body of patient safety research and practices. However, SA only recently introduced a comprehensive initiative to improve the patient safety culture in its healthcare settings. Therefore, this study was part of a large research project that was conducted in collaboration between both countries to create opportunities for enhancing practices in SA.

Therefore, the aims of this study were to: (1) investigate hospitalisations due to MRPs in adult patients with CVDs and/or DM; (2) determine the major causes and risk factors contributing to MRPs; and (3) identify the main medicines associated with MRPs in both the UK and SA.

## 2. Materials and Methods

### 2.1. Study Design and Settings

A retrospective medical record review was undertaken in two hospitals in the UK and SA. The study commenced after approval was obtained from both countries. In the UK, National Health Service ethical approval was granted by the National Research Ethics Service (NRES) committee in Greater Manchester, Manchester, UK (15 October 2012, 12/NW/0768). In SA, the study was approved by the Ethics Committee at the Saudi hospital in the Najran region on 16 August 2012. Informed consent was not obtained from the study participants as the data was analysed anonymously. The anonymisation process was verified by the medical records departments in both countries.

### 2.2. Data Collection

Data from medical records in both hospitals (UK and SA) were collected by the principal researcher (Abdullah Al Hamid) after participating in the relevant training from each hospital. The data collection was carried out at the pharmacy departments and was facilitated by liaising with the medical records departments. Thus, the researcher was able to obtain the required information from pre-anonymised medical records.

A data collection tool was developed, based on the Pharmaceutical Care Network Europe (PCNE) classification tool [4]. The tool was used to extract the following data from each medical record: demographic information, medicines upon admission, diagnosis, laboratory tests, clinical notes (including any risk factors), allergies, and changes in medication.

A total of 300 medical records (150 from each hospital) were randomly selected from all admissions between April 2012 and April 2013 in the UK hospital and between June 2012 and June 2013 in the SA hospital. This sample size was calculated using the Cochrane (1977) equation, which suggested that a minimum of 113 medical records in each country should be reviewed [13]. However, as the investigation was undertaken at two different hospitals with rather distinct features, the sample size was increased to 150 at each hospital. For the randomisation process, a computer-generated sequence technique was deployed.

The inclusion criteria were: Patients aged ≥18 years old who had been admitted with CVDs and/or DM. Patients were identified using the International Classification of Diseases (ICD-10) codes [14].

### 2.3. Definitions Used in the Study

A MRP was defined as “an event or circumstance that involves drug therapy and actually or potentially interferes with the desired health outcomes” [4,15]. This definition of MRPs included: adverse drug events (ADEs), adverse drug reactions (ADRs), and medication errors (MEs). An ADE was defined as “an injury resulting from the appropriate or inappropriate use of medicines” [16]). An ADR was defined as “any noxious, unintended and undesired effect of a drug, which occurs at doses in humans for prophylaxis, diagnosis, or therapy” [17]. A ME was defined as “the preventable mishaps occurring in the process of drug prescription, transcription, dispensing, administration, monitoring and adherence, which could lead to inappropriate use of medication or harm to the patient” [18]. Accordingly, a hospitalisation was defined as medicine-related if it was directly associated with one of the MRP categories mentioned above (*i.e.*, ADE, ADR, or ME). To determine whether the hospitalisation was due to a MRP or not, the PCNE criteria [4] were used. Thus, if two or more reviewers agreed that the medicine(s) definitely or potentially were attributable for the hospital admission, then the patients’ and MRPs’ characteristics were included to the results.

CVDs were defined as the presence of acute coronary syndrome, ischaemic heart disease (IHD), heart failure, arrhythmia, cardiomyopathy, or as stated in the medical records [19]. DM was defined as a metabolic disorder or multiple aetiologies, characterised by chronic hyperglycaemia with disturbances in carbohydrate, fat, and protein metabolism resulting from defects in insulin secretion, insulin action, or both. The effects of DM include long-term damage to various organs [2]. Medicine non-adherence was defined as “the extent to which the patient’s drug taking behaviour (in terms of taking medication) coincides with the prescription” [20]. Medicine non-adherence was detected from the medical records, either by patient declaration (definite) or by a history of non-adherence (potential). Polypharmacy was defined as the use of five or more medicines [21]. Comorbidities were defined as chronic illnesses or diseases that required long-term treatment [22].

### 2.4. Classification and Validation of MRPs

This study adopted the current PCNE-based classification of MRPs version 6 [4]. It has four primary domains for problems (P1-treatment effectiveness, P2-adverse reactions, P3-treatment costs, and P4-others). There are eight primary domains for causes (C1-drug selection, C2-drug form, C3-dose selection, C4-treatment duration, C5-drug use/administration process, C6-logistics, C7-patient, and C8-other) [4]. Moreover, the MRPs’ level of harm was measured using the scale of harm developed by the National Patient Safety Agency (NPSA) [23]. The NPSA scale classifies the harm resulting from MRPs into four categories: no harm, low, medium, or severe. Assignment of these depended on the effect of the MRP(s) on patient safety.

The medical records included in this study were reviewed by two expert panels (one in each country). Each panel consisted of a doctor and three pharmacists who: had a minimum experience of 10 years, had specific expertise in analysing patient safety incidents, and were available for 1–2 days per week. The reviewers received training on how to use the PCNE tool, utilising standard patients’ medical records. The study protocol, definitions, and examples of MRPs were discussed during the training. Furthermore, prior to the main study, the reviewers from each expert panel conducted a pilot study to ensure that they fully understood how to use the tools and forms. Reviewers within each team were able to consult each other and the principal researcher in case of a doubt. After reaching 80% agreement for the pilot study results, the reviewers commenced with the main study. Then, the reviewers on each expert panel reviewed 20% of the total included medical records in each country. The outcomes of the review of each record was: (1) no MRP; (2) definite MRP; or (3) potential MRP. The level of agreement between the experts and the researcher was assessed using the kappa coefficient [24]. In both sides, moderate to substantial agreement was obtained.

### 2.5. Data Analysis

Data analysis was carried out using IBM SPSS version 21.0 (SPSS Inc., New York, NY, USA). Descriptive statistics were applied to the following characteristics: MRPs, patient demographics, risk factors, comorbidities, medicines, and medicine classes. Parameters within each characteristic (demography, comorbidities, risk factors, medicines, and medicine classes) were assessed for all patients. Parameters were used in a binary logistic regression to assess their interaction with the MRPs. More specifically, the binary logistic regression investigated the ability of each of the parameters to predict the presence or absence of MRPs in order to calculate the odds ratio (OR) between the individual parameters and the prevalence of MRP.

## 3. Results

### 3.1. Demographic Characteristics of the Study Population

The study included 300 patients from both the UK and SA. Table 1 shows the patients’ condition, duration of stay, referral source, age, gender, marital status, ethnicity, language, and religion. There were no significant differences between patients in both countries in terms of condition, duration of stay, age, gender, marital status, ethnicity, and language (*p* > 0.05). On the other hand, referral source (*p* = 0.019) and religion (*p* = 0.003) were significantly different between the UK and SA.

Regarding condition, the majority of the investigated patients had only CVD or both CVD and DM; fewer patients suffer from DM only. In addition, most of the patients (59%) had a hospital stay of five days or less. The main age group of the patients was in the range of 41–85 years. Men were more prevalent (>50%) in both countries. On the other hand, marital status, ethnicity, and language were slightly different between both countries. In the UK, most of the patients were of unknown marital status, of white British ethnicity, and spoke English. However, most of the patients in SA were married, of Arabic ethnicity, and spoke Arabic. Additionally, significant differences were observed between patients in both countries in relation to the source of referral. In SA, all patients were admitted through A/E departments; whereas, patients in the UK were admitted through seven different departments. Hence, the UK had a wide range of referral despite that the majority of the patients came from A/E. Similarly, religion was significantly different between patients in both countries—over 50% of the UK patients were Christian, and 97% of SA patients were Muslims.

The results of the logistic regression showed variations in the results between the UK and SA (Table 2). In the UK the positive predictors for MRPs were duration of stay, gender, and religion. None of the aforementioned factors was significantly associated with MRPs, though. On the other hand, in SA the positive predictors were age, marital status, ethnicity, and religion. Among these factors, only age was significantly associated with MRPs (OR = 1.03 (1–1.05), *p* = 0.024).

### 3.2. Prevalence of MRPs

There were 197 (66%) MRPs identified in this study (Supplementary Tables S1 and S2), including 103 (52.2%) in the UK and 94 (47.7%) in SA (Table 3). Of these MRPs, 58.7% and 41.5% resulted in hospitalisation in the UK and SA, respectively. There was no significant difference in the MRP types between both countries (*p* = 0.68). Thus, definite MRPs represented 70% and 67% of the total MRPs encountered in both the UK and SA, respectively. Likewise, there was no significant difference in the type of MRP problem (as defined by PCNE classification), cause (as defined by PCNE classification), and level of harm (*p* > 0.05) between both countries. Thus, the key MRP problems encountered were treatment effectiveness (TE) and ADRs. These two types or problems accounted for 90.0% of the total problems in the UK and 97.8% of the total problems in SA. In addition, the main reported causes were drug selection, dose selection, and patient non-adherence. Other causes that were documented in the UK and not in SA included drug-alcohol interaction, treatment duration, drug use/administration, drug form, drug misuse/abuse, and logistics. Furthermore, in most cases MRPs caused a low or moderate level of harm; MRPs caused severe harm in less than 9% of patients in both countries.

### 3.3. Comorbidities Associated with MRPs

A total of 106 patients had comorbidities: 58 in the UK and 48 in SA. In both the UK and SA, hypertension (HTN) was the most prevalent comorbidity (85 patients in the UK and 84 patients in SA). This was followed by diabetes mellitus type 2 (DMT2), present in 68 patients (45.0%) and 25 patients (16.6%) in SA and the UK, respectively. Additional comorbidities reported in the UK sample included hypercholesterolemia and atrial fibrillation (AF), which were reported in 25 (16.6%) and 24 (16.0%) patients, respectively. IHD was reported in 34 (30.0%) patients in SA.

The association of these comorbidities with MRPs was confirmed by the results of the binary logistic regression (Table 2). HTN and DMT2 showed an association with MRPs in both the UK and SA. Additionally, AF, asthma, and IHD were predictors for MRPs in the UK, and NSTEMI was found to be a predictor for MRPs in SA.

### 3.4. Risk Factors Associated with MRPs

There was no significant difference between the prevalence of risk factors contributing to MRPs in both countries (*p* = 0.1918). Polypharmacy was the major risk factor associated with MRPs. It was reported in 50.9% of the cases in the UK and 40.9% of the cases in SA (Table 4). This was followed by patient non-adherence, impaired kidney function, smoking, impaired liver function, and obesity. Moreover, certain risk factors were only reported in the UK, including dependent living situation, impaired cognition, HTN, excessive caffeine intake, anaemia, family history of CVDs, and stress. Furthermore, four risk factors were only reported in SA: depression, epilepsy, smoking history, and anorexia.

Logistic regression results confirmed four predictors for MRPs in the UK and three in SA. The risk factors associated with MRPs in the UK were patient non-adherence, impaired kidney function, obesity, and anaemia. The risk factors associated with MRPs in SA were impaired liver function, smoking, and obesity. However, none of these predictors were significantly associated with MRPs.

### 3.5. Medicines Involved in MRPs

Different medicines were involved in the reported MRPs in both countries. In the UK, medicines used for CVDs contributed to a higher number of MRPs than diabetic medicines. On the other hand, diabetic medicines were involved in a higher number of MRPs in SA.

In the UK, medicines for CVDs contributed to 83 MRPs. Of these, 36 were associated with antihypertensive medicines (*i.e.*, angiotensin converting enzyme inhibitors (ACEI), diuretics, calcium channel blockers (CCB), angiotensin receptor antagonists (AT-antagonist), alpha-blockers, and centrally acting agents), 15 were used for anti-platelet activities (aspirin and clopidogrel), 15 were antiarrhythmics (beta blockers and digoxin), seven were anticoagulants (warfarin), and seven were lipid-regulating drugs (statins). In addition, antidiabetic medicines were implicated in 21 MRPs, of which oral antidiabetic agents were implicated in 10 MRPs and insulin was implicated in 11 MRPs.

In SA, antidiabetic medicines contributed to the majority of the MRPs. Insulin was implicated in 44 MRPs while oral antidiabetic agents were associated with five MRPs. Nonetheless, 32 MRPs were related to CVD medicines, including antihypertensive (*i.e.*, ACEIs, CCBs), anticoagulants (aspirin), antiarrhythmic (beta blockers and digoxin), and antihyperlipidemics (statins).

Logistic regression results showed that four medicine classes were predictors of MRPs in the UK: and were antiarrythmics (beta-antagonists), two antiHTN medicine classes (ACEIs and potassium sparing diuretics/aldosterone antagonists), as well as antisecretory and mucosal protective agents (PPIs). More medicine classes (*n* = 10) were associated with MRPs in SA and included: antianginals (nitrates), antiarrhythmics (beta-antagonists), antidiabetics (sulfonyl urea derivatives), four antiHTN medicine classes (AT-receptor antagonist, CCB, potassium spairing diuretics/aldosterone antagonists, loop diuretics), antiplatelets, insulin, and lipid-regulating drugs (statins). Of the aforementioned medicine classes, only insulin was found to be significantly associated with MRPs (OR = 3.85 (1.55–9.55), *p* = 0.004).

## 4. Discussion

To the best of our knowledge, this is the first study that investigated hospitalisations resulting from MRPs in adult patients with CVDs and DM in both the UK and SA. Four similar studies investigating CVD and/or DM have been reported in the literature. Two of these studies investigated MRPs in patients with CVDs in Ethiopia [25] and India [26]. The remaining two studies investigated hospitalisation due to MRPs in patients with DM [27] and patients with HTN/DMT1 in Malaysia [28].

Our study indicated that MRPs are a major health issue that leads to hospitalisation in both the UK and SA. MRPs were present in more than 50% of the total study cohort (58.7% in the UK and 52.6% in SA). Out of the identified MRPs, 70.9% and 41.5% led to hospitalisation in the UK and SA, respectively. However, fewer than 10% of these MRPs were severe cases. Four similar studies in the literature reported variable percentages of MRPs: 11.0% [27], 32.8% [25], 47.0% [26], and 90.5% [28]. The variation in the percentages of MRPs between the studies could be attributed to the variation in the study characteristics and the patients included in the samples. Thus, the first three studies considered patients with either a diagnosis of CVD or DM, whereas the latter study investigated patients with coexisting HTN and DM [20]. Our results were closer to the study conducted by Zaman-Huri *et al.* [28] for two reasons: (1) similarity in the study sample size, and (2) definitions and methodology used.

It is worth noting that this study was not initially designed to compare the differences between the two countries. Nonetheless, any encountered variations could be explained by the fact that both countries have different healthcare systems and population characteristics, which cannot be controlled for to allow for direct comparisons.

### 4.1. Problems and Causes

This study revealed that the main MRP problems were related to lack of TE and ADR. However, there were differences in the problems between both countries. The UK had high representation of ADRs (45.2%), while MRPs in SA were primarily due to lack of TE (70%). This data agreed with two similar studies in India [26] and Ethiopia [25], which reported a lack of TE as the main MRP problem encountered. In SA, issues such as a lack of a qualified healthcare workforce, absence of national clinical guidelines, and lack of communication between healthcare providers can all contribute to poor TE [29,30,31].

A similar situation was observed with the causes of MRPs. Drug and dose selection accounted for 70% of the causes of the MRPs in SA while ADRs were the main cause of MRPs in the UK. In the UK, this could be attributed to the overuse of medicines as preventive interventions for chronic conditions. On the other hand, the quality of the healthcare system at public hospitals in SA [32] is influenced by several factors: limited finances coupled with high demand for free services, poor access to some healthcare facilities, lack of use of electronic healthcare strategies, absence of a national policy, and a lack of national health information—all of which might be the main reasons for the occurrence of ME [32]. These issues were identified despite the fact that the Saudi healthcare system was ranked 26th out of the 190 global health systems in 2000 [33].

### 4.2. Comorbidities

In both the UK and SA, HTN and DMT2 were the major comorbidities associated with MRPs. Patients with HTN and/or DMT2 are often on polypharmacy—this could increase the possibility of ADRs, drug-drug interactions, or even patients forgetting to take medicines [34,35]. In this study, multiple CVDs were found to correlate with MRPs, including: AF, congestive heart failure, IHD, left ventricular disease, myocardial infarction (MI), and supraventricular disease.

### 4.3. Risk Factors

Patient non-adherence and polypharmacy were the main risk factors in both the UK and SA. In this respect, the presence of multiple medicines and their associated ADRs may have led to patients’ non-adherence to the treatment regimens. Both risk factors have been acknowledged in other studies, where a high number of medicines taken by patients were found to lead to drug-drug interactions [26]. Additionally, obesity and impaired liver function were major risk factors in SA, which can be attributed mainly to the patients’ sedentary lifestyle. In addition, unbalanced diet (*i.e.*, excessive fat and carbohydrate intake) was a major issue, especially in CVD and diabetic patients in SA. This diet might result in poor control over the conditions and drug-food interactions that interfere with the effect of the medicines [36].

On the other hand, patient non-adherence to therapy was higher in the UK (14%) than SA (6%). Similar studies reported patient non-adherence in the UK as a major concern; studies revealed that patients tend to assume medicines are pharmaceutical toxins and believe they are harmful [1,34,37,38], and that multiple regimens are given to patients with chronic diseases. On the other hand, in SA it was difficult to fully investigate patients’ non-adherence as this information was not a compulsory part of the medical record, so it is assumed that the percentage reported in this study does not reflect the actual extent of the phenomenon and that non-adherence remains underreported [39].

### 4.4. Medicines Involved

Medicines that were frequently associated with MRPs included insulin, antihypertensives, antiarrhythmics, and lipid-regulating agents. Insulin had a higher association with MRPs in SA than in the UK. This was partially affected by SA patients’ diet, where patients needed dose adjustment for insulin depending on their food consumption, especially during religious events such as Ramadan and Hajj. Nevertheless, in both the UK and SA, antihypertensive, antiarrhythmic, and lipid-regulating medicines had a significant correlation with MRPs, mainly due to ADRs and dose/regimen issues. The most reported ADRs were epigastric pain/bleeding due to aspirin and cough due to ACEIs. Moreover, the highest TE problems were associated with incorrect doses of atenolol resulting in tachycardia/bradycardia. These findings are consistent with other studies that showed these specific medicines were often involved in MRPs [28].

### 4.5. Strengths and Limitations

This study highlighted the need to review the clinical guidance in both countries, especially when both conditions (CVDs and DM) coexist. Furthermore, it shed light on the significance of developing a screening tool that includes quantitative and qualitative variables (risk factors) to predict and identify patients at higher risk of MRPs prior to admission.

This study had some limitations. (1) The studied conditions, use of multiple sources of information, and time limitations did not allow for a prospective study design. Therefore, a retrospective design was used, which included the patients’ medical record indication of diagnosed conditions, emergency and progression notes, laboratory results, and prescriptions; (2) the sample size was relatively small (*n* = 300) due to the limited time frame of the study. However, the sample size was statistically powered and comparable to other studies in the literature; (3) the study was based on information documented in the medical record and it was assumed that this information was accurate.

Finally, although this study estimated the scale of the problem, these estimates need to be interpreted carefully as our analyses were limited to data from two hospitals and the generalisability of the results cannot be guaranteed. Several factors could have led to under/over-estimation of MRPs, including: (1) the recruited cohorts may not be representative of the general populations in both countries; (2) the study sample is relatively small compared to the number of patients with CVDs and DM, and (3) different treatment regimens were used in both countries, which may have affected the prevalence and types of MRPs encountered.

## 5. Conclusions

The study showed that MRPs were a major issue that could lead to hospitalisation and were prevalent in more than half of the study participants in the UK and SA. The severity of the identified MRPs was low in both countries, and severe cases accounted for less than 10% of all cases. Although both cohorts experienced similar MRPs, the leading problem of MRPs was ADR in the UK and lack of TE in SA. The major causes for MRPs in both countries were ADRs and drug/dose selection. Moreover, polypharmacy and patient non-adherence were the main risk factors associated with MRPs. Additionally, the main medicines involved in MRPs were insulin and antihypertensive agents. Therefore, due to the complexity of treating and managing these conditions, additional training should be given to both healthcare professionals and patients to avoid MRPs and the subsequent unnecessary hospital admissions that affect both governments and societies worldwide.

## Figures and Tables

**Table 1:** Demographic information of the patients included in this study.

Parameter	UK (N (%))	SA (N (%))
**Condition**		
CVD	97 (64.5)	69 (46)
DM	11 (7.33)	0 (0)
CVD/DM	42 (28)	81 (54)
**Duration of stay (days)**		
1–5	89 (59.3)	108 (72)
6–10	35 (23.3)	36 (24)
11–15	15 (10)	3 (2)
16–20	3 (2)	1 (0.67)
>21	8 (5.33)	2 (1.33)
**Source of referral**		
A/E	138 (92)	150 (100)
Others ^1^	12 (8)	0 (0)
**Age (years)**		
≤25	2 (1.33)	6 (4)
25–40	10 (6.67)	16 (10.7)
41–55	15 (10)	43 (28.7)
56–70	46 (30.7)	54 (36)
71–85	58 (38.7)	24 (16)
86–100	19 (12.7)	7 (4.65)
**Gender**		
Female	64 (42.7)	46 (30.7)
Male	86 (57.3)	104 (69.3)
**Marital status**		
Married	28 (18.7)	142 (94.7)
Widow	3 (2)	0 (0)
Unknown	109 (72.7)	0 (0)
Single	7 (4.67)	7 (4.67)
Divorced	3 (2)	1 (0.67)
**Ethnicity**		
Arabic	0 (0)	139 (92.7)
White British	107 (71.3)	0 (0)
Pakistani	13 (8.67)	4 (2)
Bangladeshi	3 (2)	4 (2.67)
Indian	6 (4)	3 (2)
Others ^2^	27 (18)	0 (0)
Not specified	8 (5.33)	0 (0)

≤: less than or equal, A/E: Accident and Emergency, CVD: cardiovascular disease, DM: Diabetes Mellitus, GP: general practitioner, N: number, OP: operation, SA: Saudi Arabia. ^1^: A/E, OP, GP, other hospital, residence, cardiology clinic. ^2^: Black, white (Irish and others), Filipino).

**Table 2:** Potential predictors for MRP occurrence in both countries.

Criteria	UK (OR)	*p*-Value	SA (OR)	*p*-Value
	**Demography**			
Duration of stay	1.05 (0.98–1.11)	0.163	0.96 (0.88–1.05)	0.403
Patient age (years)	0.99 (0.97–1.02)	0.534	1.03 (1–1.05)	0.024
Patient gender	1.64 (0.78–3.41)	0.190	0.78 (0.38–1.62)	0.508
Marital status	0.96 (0.64–1.44)	0.845	1.16 (0.29–4.52)	0.835
Ethnicity	0.98 (0.82–1.16)	0.785	1.08 (0.52–2.23)	0.831
Religion	1.04 (0.89–1.22)	0.619	1.75 (0.38–8.13)	0.478
	**Risk Factors**			
Polypharmacy	0.93 (0.39–2.23)	0.876	0.78 (0.38–1.61)	0.502
Patient non-adherence	3.48 (0.87–13.9)	0.079	0	0
Impaired liver function	0.71 (0.15–3.32)	0.663	1.94 (0.33–11.4)	0.461
Impaired kidney function	1.22 (0.48–3.09)	0.679	0.55 (0.19–1.57)	0.265
Smoking	0.79 (0.19–3.25)	0.738	1.22 (0.42–3.54)	0.714
Obesity	1.77 (0.1–32.6)	0.7	1.82 (0.57–5.74)	0.31
Anaemia	2.45 (0.15–23.8)	0.439	0	0
	**Comorbidities**			
AF	0.88 (0.35–2.22)	0.784	1.08 (0.15–7.53)	0.94
Asthma	0.84 (0.25–2.86)	0.783	2.72 (0.42–17.6)	0.293
DMT2	1.25 (0.57–2.77)	0.577	3.99 (1.87–8.53)	0.001
HTN	1.44 (0.7–2.96)	0.32	1.02 (0.47–2.22)	0.951
IHD	0.49 (0.18–1.33)	0.16	1.33 (0.49–3.65)	0.577
NSTEMI	1.01 (0.33–3.08)	0.993	0	0
	**Medicine Classes**			
Antiangina (nitrates)	0.88 (0.35–2.23)	0.794	1.39 (0.58–3.39)	0.461
Antiarrhythmic (beta-antagonists)	1.11 (0.53–2.32)	0.788	1.07 (0.44–2.59)	0.886
Antidiabetic (sulfonyl urea derivatives)	0	0	2.75 (0.49–15.3)	0.248
AntiHTN (ACEIs)	1.01 (0.48–2.12)	0.987	0.63 (0.29–1.36)	0.237
AntiHTN (AT-receptor antagonist)	0	0	3.48 (0.85–14.3)	0.083
AntiHTN (CCB)	0	0	2.25 (0.66–7.71)	0.108
AntiHTN (potassium spairing diuretics/aldosterone antagonists)	1.44 (0.51–4.08)	0.487	1.19 (0.37–3.89)	0.763
AntiHTN (loop diuretics)	0	0	1.31 (0.5–3.39)	0.583
Antiplatelets	0.39 (0.17–0.88)	0.023	1.31 (0.2–8.54)	0.776
Antisecretory and mucosal protective agents (PPIs)	1.39 (0.65–2.96)	0.398	0.64 (0.19–2.04)	0.448
Insulin	0	0	3.85 (1.55–9.55)	0.004
Lipid regulating drugs (statins)	0.56 (0.24–1.28)	0.168	1.37 (0.54–3.48)	0.507
	**Medicines**			
Amlodipine	0	0	2.34 (0.59–9.35)	0.229
Aspirin	0.22 (0.02–2.12)	0.19	1.09 (0.35–3.42)	0.889
Atenolol	0	0	7.79 (0.98–62.1)	0.052
Bisoprolol	2.87 (0.43–19.1)	0.275	0.2 (0.02–2.23)	0.203
Carvediolol	0	0	1.16 (0.26–5.12)	0.843
Clopidogrel	3.75 (0.41–34.6)	0.783	0.51 (0.19–1.38)	0.187
Enoxoparin	0	0	1.19 (0.41–3.5)	0.742
Furosemide	18.7 (0.98–357)	0.052	1.58 (0.5–4.95)	0.434
Glibenclamide	0	0	3.22 (0.47–21.9)	9.232
Insulin	0	0	8.51 (2.84–25.5)	0.001
Isosorbide mononitrate	5.11 (0.41–63.8)	0.206	2.09 (0.74–5.86)	0.163
Lisinopril	0	0	1.52 (0.51–4.56)	0.456
Losartan	0	0	7.58 (0.79–72.9)	0.079
Metoprolol	0	0	1.45 (0.52–4.07)	0.476
Omeprazole	1.05 (0.1–11.5)	0.968	0.52 (0.11–2.5)	0.413
Simvastatin	0.02 (0–0.49)	0.017	1.18 (0.35–3.97)	0.793

ACEI: angiotensin converting enzyme inhibitor, AF: atrial fibrillation, AT-receptor antagonist: angiotensin receptor antagonist, CCB: calcium channel blocker, CHF: congestive heart failure, CVD: cardiovascular diseases, DM: diabetes, DMT2: diabetes mellitus type 2, GTN: glyceryl trinitrate, HTN: hypertension, IHD: ischaemic heart disease, LVD: left ventricular disease, MI: myocardial infarction, NSTEMI: non-ST elevated myocardial infarction, OP: Operation, OR: odds ratio, PPI: proton pump inhibitor, SVD: supraventricular disease.

**Table 3:** Medicine-related problems’ prevalence and characteristics in each country.

Parameter	UK (N (%))	SA (N (%))
MRPs		
Definite	72 (69.9)	63 (67)
Potential	31 (30.1)	31 (33)
Hospitalisation		
MRPs	88 (58.7)	63 (41.5)
Problem		
ADR	47 (45.2)	19 (20.4)
ME	4 (3.8)	3 (2.15)
TE	47 (45.2)	72 (77.4)
Others	5 (4.8)	0 (0)
Causes		
ADR	47 (45.2)	19 (20.4)
Drug selection	23 (23.1)	29 (31.2)
Dose selection	20 (19.2)	24 (25.6)
Patient non-adherence	14 (13.5)	7 (6.45)
Others *	7 4.67)	0 (0)

ADR: adverse drug reaction, ME: medication error, MRP: medicine related problems, TE: treatment effectiveness. Others *: drug-alcohol interaction, Drug overdose, treatment duration, drug use, drug form and logistics.

**Table 4:** The reported risk factors from both countries.

Parameter	UK (N (%))	SA (N (%))
Polypharmacy	111 (50.9)	106 (40.4)
Independent living situation	8 (3.67)	0 (0)
Lack of physical movement	11 (5.05)	1 (0.38)
Patient non-adherence	11 (5.05)	3 (1.14)
Alcohol consumption	4 (1.85)	1 (0.38)
Impaired liver function	10 (4.59)	6 (2.29)
Impaired cognition	10 (4.59)	0 (0)
Impaired kidney function	25 (11.5)	18 (6.86)
Smoking	11 (5.05)	16 (6.1)
Obesity	6 (2.75)	15 (5.71)

CVDs: cardiovascular diseases, N: number.

## Supplementary Materials

The following are available online at www.mdpi.com/1660-4601/13/5/479/s1, Table S1: Characteristics of MRPs (*n* = 103) encountered in the UK study, Table S2: Characteristics of MRPs encountered in the SA study.

## Abbreviations

The following abbreviations are used in this manuscript:

A/E	Accident and emergency
ACEI	Angiotensin converting enzyme inhibitor
ADE	Adverse drug event
ADR	Adverse drug reaction
AF	Atrial fibrillation
AT	Angiotensin
CCB	Calcium channel blocker
CHF	Congestive heart failure
CVDs	Cardiovascular diseases
DM	Diabetes mellitus
DMT2	Diabetes mellitus type 2
GP	General practitioner
GTN	Glyceryl trinitrate
HTN	Hypertension
ICD-10	International Classification of Diseases
LVD	Left ventricular disease
IHD	Ischaemic heart disease
ME	Medication error
MI	Myocardial infarction
MRH	Medicine-related hospitalisation
MRP	Medicine-related problem
N	Number
NCDs	Non-communicable diseases
NICE	National Institute of Health and Care Excellence
NPSA	National Patient Safety Agency
NSTEMI	Non-ST elevated myocardial infarction
OP	Operations
OR	Odds ratio
PCNE	Pharmaceutical Care Network Europe
PPI	Proton pump inhibitor
SA	Saudi Arabia
SVD	Supraventricular disease
TE	Treatment effectiveness
WHO	World Health Organisation
