# Supplementary material for: Hospitalisation Resulting from Medicine-Related Problems in Adult Patients with Cardiovascular Diseases and Diabetes in the United Kingdom and Saudi Arabia
Source: Int J Environ Res Public Health. 2016 May 9;13(5):479. doi: 10.3390/ijerph13050479 (PMC4881104; doi:10.3390/ijerph13050479)
Supplement: Supplementary file 1 [file ijerph-13-00479-s001.pdf]

Supplementary Materials: Hospitalisation Resulting from Medicine-Related Problems in Adult Patients with Cardiovascular Diseases and Diabetes in the United Kingdom and Saudi Arabia

Abdullah Al Hamid, Zoe Aslanpour, Hisham Aljadhey and Maisoon Ghaleb

Table S1. Characteristics of MRPs (n = 103) encountered in the UK study.

Patient Number	Type	Severity	MRP	*N*	Medicine Involved	Cause of Admission
1	P	Moderate	Chest pain, nausea and vomiting, sweating, Hyperkalaemia, continuous cough	1	ACEI (lisinopril 10 mg)	Yes
2	P	Low	Chest pain (due to unregulated dose, troponin level was too high)	1	Aspirin 75 mg	Yes
3	D	Moderate	High blood sugar level	1	Novomix 30 (short acting insulin)	Yes
4	D	Moderate	High blood sugar level, vomiting	1	Humulin M3 (Intermediate and long acting insulin)	Yes
5	D	Moderate	Hyperglycaemia Cough	2	Olanzapine 5 mg<br>Ramipril 1.25 mg	No
6	D	Moderate	Uncontrolled blood pressure, Headache, palpitation	1	Telmisartan 80 mg<br>Doxazosin 8 mg	No
7	P	Moderate	Continuous high blood pressure, rising troponin level due to medication cardiotoxicity	2	Amlodipine 10 mg<br>Clopidogrel 75 mg<br>Aspirin 75 mg (?)	No
8	P	Low	Hyperglycaemia	1	Lantus insulin 26U	No
9	P	Low	High BM	1	Lantus insulin 26U SC<br>Novorapid SC	Yes
10	D	Moderate	Polyurea and polydipsia, ketoacidosis	1	Metformin 1 g<br>Novorapid 4–8 u/day and 10–15 u at lunch or dinner<br>Lantus 28 u at evening	Yes
11	P	Low	drowsiness, fall and head injury, confusion, dehydration and tachycardia	1	methadone 50 mL<br>dihydrocodeine 30 mg	Yes
12	D	Moderate	Hyperglycaemia	1	Gliclazide 80 mg	Yes
13	p	Low	Near syncope, hypertension	1	Doxazosin 8 mg<br>Amlodipine 10 mg<br>Atorvastatin 80 mg<br>Ramipril 10 mg	Yes
14	D	Moderate	Hypertension, headache, dizziness	1	Temisartan 40 mg	Yes
15	D	Moderate	Angina, chest pain at rest, heavy ache down the left arm, tightness of jaw, nausea and vomiting	1	Nitrglycerine<br>Isosorbide mononitrate	Yes
16	D	Moderate	Chest pain troponin negative	1	Isosorbide mononitrate	Yes

Table S1. *Cont*.

Patient Number	Type	Severity	MRP	*N*	Medicine Involved	Cause of Admission
17	P	Moderate	Mild chest discomfort and exertion, Troponin level high	1	Aspirin 75 mg	Yes
18	P	Low	Chest pain, troponin level low	1	Nitroglycerine	Yes
19	D	Moderate	Melena	1	Aspirin 75 mg	Yes
20	D	Severe	PR bleeding and abdominal pain	1	Methotrexate 12.5 mg	Yes
21	D	Severe	Melena	1	Aspirin 75 mg<br>Clopidogrel 75 mg	Yes
22	D	Moderate	Deranged liver function test	1	Atorvastatin 40 mg	No
23	D	Moderate	Hypotension	1	Furosemide 40 mg<br>Ramipril 2.5 mg<br>Spironolactone 25 mg	Yes
24	D	Severe	Bleeding<br>Confusion (potential)<br>Antibiotic resistance (potential)<br>Hypotension	4	Warfarin 3 mg/4 mg<br>Ferrous fumarate<br>Digoxin 62.5 mg<br>Quinine sulfate 300 mg<br>Furosemide 40 mg<br>Amlodipine 5 mg<br>Spironolactone 25 mg	No
25	D	Low	Melena	1	Aspirin 75 mg	No
26	D	Low	Anaemia, chronic kidney disease, fluid overload which resulted in pulmonary oedema	2	Ferrous fumarate<br>Furosemide	Yes
27	D	Low	Hypercalcemia	1	Calcium	No
28	D	Moderate	SOB, palpitation, abnormal PTINR	2	Bisoprolol 5 mg<br>Warfarin 12 mg	Yes
29	P	Low	Cough	1	Simvastatin 40 mg	Yes
30	D	Severe	Haemoptysis	1	Warfarin	Yes
31	D	Moderate	Palpitations	1	Amlodipine 5 mg	Yes
32	D	Moderate	Ankle swelling	1	Amlodipine 10 mg	Yes
33	P	Low	Oedema	1	Furosemide 20 mg	Yes
34	D	Moderate	SOB	1	Digoxin	Yes
35	D	Moderate	Hypoglycaemia	1	Insulin	Yes
36	D	Moderate	High blood pressure<br>Hyperglycaemia<br>Diarrhoea	3	Candesartan 4 mg<br>Insulin<br>Amoxicillin	Yes
37	D	Moderate	High BM<br>Glycosuria	2	Metformin 850 mg<br>Gliclazide 160 mg<br>Pioglitasone 45 mg	Yes

Table S1. *Cont*.

Patient Number	Type	Severity	MRP	*N*	Medicine Involved	Cause of Admission
38	D	Moderate	Glycosuria	1	Gliclazide 160 mg<br>Metformine 1 g<br>Humulin mix 25	Yes
39	D	Moderate	Hyperglycaemia	1	Metformin 500 mg	Yes
40	D	Moderate	Hyperglycaemia	1	Insulatard	Yes
41	D	Moderate	Hyperglycaemia	1	Glimipiride	Yes
42	P	Moderate	Renal failure	1	Spironolactone	Yes
43	D	Moderate	OR bleeding due to aspirin	1	Aspirin	Yes
44	D	Moderate	GORD	1	Aspirin	Yes
45	D	Moderate	Chest pain, SOB	2	Aspirin<br>GTN	Yes
46	D	Moderate	Sinus bradycardia	1	Bisoprolol	Yes
47	p	Moderate	SOB, palpitation,<br>abnormal PTINR	1	Aspirin	Yes
48	D	Low	worsening heart failure	1	Furosemide 40 mg<br>Ramipril 1.25 mg	Yes
49	P	Moderate	Angina	1	Asassantin retard	Yes
50	D	Moderate	Impaired kidney function	1	Ramipril	Yes
51	D	Moderate	Hypoglycaemia	1	Gliclazide 80 mg	Yes
52	D	Moderate	Gastroenteritis	1	Aspirin 75 mg<br>Prednisolone 5 mg	Yes
53	P	Moderate	Stroke	1	Aspirin 75 mg	Yes
54	D	Severe	SOB, chest pain, sweating	1	Disopyramide 300 mg	Yes
55	D	Moderate	aortic stenosis contraindication/<br>risk of hypotension	1	Isosorbide mononitrate	No
56	P	Moderate	palpitation<br>atrial fibrillation	1	Losartan potassium<br>100 mg	Yes
57	D	Moderate	NSTEMI<br>HF	2	Atorvastatin 20 mg<br>Ramipril 1.25 mg	Yes
58	D	Moderate	SOB in asthmatic patient<br>Low systolic BP	2	Propranolol 20 mg<br>Indapamide 2.5 mg<br>Nifedipine 40 mg<br>Moxonidine 30 mg	Yes
59	D	Low	Diarrhoea	1	Tiotropium 18 mcg<br>Aminophylline 450 mg<br>Salbutamol	Yes

Table S1. *Cont*.

Patient Number	Type	Severity	MRP	*N*	Medicine Involved	Cause of Admission
60	D	Moderate	Syncope	1	Glyceryl trinitrate	Yes
61	P	Severe	SOB on exertion associated with chest pain	1	Patient stopped taking his medication	Yes
62	P, D	Moderate	ACS<br>Bradycardia	2	Ticagrelor 90 mg<br>Bisoprolol 1.25 mg	Yes
63	P	low	ALP (Liver function tests)<br>Eye sight loss	2	Ezetimibe 10 mg<br>Ramipril 2.5 mg<br>Sotalol 40 mg	Yes
64	P	low	Chest pain	1	Bisoprolol 1.25 mg<br>Atorvastatin 80 mg<br>Ramipril 1.25 mg	Yes
65	D	low	Kidney function (low creatinine)	1	Ramipril 1.25 mg	No
66	P	low	Chest pain	1	Simvastatin 40 mg<br>Lisinopril 10 mg	Yes
67	P	low	Chest pain	1	Simvastatin 40 mg<br>Ramipril 7.5 mg	Yes
68	P	low	Chest pain	1	Simvastatin 40 mg<br>Ramipril 2.5 mg	Yes
69	P	low	Bleeding ulcer	1	Aspirin 75 mg<br>Ferrous fumarate 305 mg	No
70	P,P,D	Moderate	Increased INR<br>Diarrhoea<br>Nausea, dizziness and weakness	3	Warfarin<br>Antibiotic<br>Warfarin and bisoprolol 1.25 mg	Yes
71	D	low	Right leg swelling	1	Hydrocortisone	Yes
72	D	Moderate	Pulmonary embolism	1	Warfarin 8.5 mg	Yes
73	D	Moderate	Pulmonary embolism	1	Warfarin 8.5 mg	Yes
74	D	Low	Hallucination	1	Madopar 62.5 mg	No
75	P	low	Tightness of chest and tachycardia	1	Glyceryl trinitrate	Yes
76	P	low	Tachycardia	1	Salbutamol TT	Yes
77	P	low	Dizziness and low blood pressure	1	Bisoprolol 10 mg	Yes
78	D	low	Sinus rhythm	1	Digoxin	Yes
79	D	low	Tachycardia	1	Sotalol 80 mg	Yes
80	D	low	Tachycardia	1	Sotalol 80 mg	Yes
81	D	low	Hypoglycaemia	1	Dexamethasone	Yes
82	D	low	Hypotension	1	Atenolol 50 mg	No
83	D	low	Palpitation	1	Lercandipine 10 mg	Yes

Table S1. *Cont.*

Patient Number	Type	Severity	MRP	*N*	Medicine Involved	Cause of Admission
84	D	low	Palpitation	1	Doxazosin 4 mg<br>Amlodipine 5 mg	Yes
85	D	low	Palpitation	1	Propafenone 150 mg	Yes
86	D	low	Palpitation	1	Digoxin 125 mcg<br>Bisoprolol 7.5 mg	Yes

Table S2. Characteristics of MRPs encountered in the SA study.

Patient Number	Type	Severity	MRP	*N*	Medicine	Cause of Admission
1	D<br>D	L<br>L	Hyperglycaemia<br>High blood pressure	2	Lisinopril 10 mg<br>Lercanidipine 20 mg<br>Simvastatin 40 mg	Yes<br>Yes
2	P<br>P	L<br>L	Hyperglycaemia<br>Hyperglycaemia	2	Insulin mix<br>Metformine 500 mg	Yes<br>Yes
3	P	S	Acute coronary syndrome	1	Simvastatin 20 mg	Yes
4	D	S	Hyperglycaemia	1	Humulin M3	Yes
5	P	L	Hyperglycaemia	1	Missing medicine	No
9	P<br>P	L<br>L	Hyperglycaemia<br>HTN	2	Novomix 30<br>Amlodipine 10 mg<br>Bisoprolol 1.25 mg<br>Ramipril 10 mg	No<br>No
10	P	L	Hyperglycaemia	1	Metformin 500 mg	No
11	P	L	Hyperglycaemia	1	Insulin mix	No
12	P	L	Hyperglycaemia	1	Metformin 1 g<br>Insulin rapid	No
13	D	L	Hyperglycaemia	1	Insulin rapid	Yes
14	D	S	Gastric bleeding	1	Aspirin 81 mg	Yes
15	P	L	Hyperglycaemia	1	Humalog mix 50:40 units<br>Metformin 2 g	No
16	P	L	Hyperglycaemia	1	Insulin mix	No
17	P	L	Hyperglycaemia	1	Insulin mix<br>Metformin 500 mg	No
18	P	L	Anaemia	1	Iron	No
19	D	L	Cough	1	Lisinopril 10 mg	Yes

Table S2. *Cont*.

Patient Number	Type	Severity	MRP	*N*	Medicine	Cause of Admission
20	D	S	Sweating, nausea and vomiting increasing in intensity	1	Levothyroxine 50 micrograms	Yes
21	D	L	HTN	1	Bisoprolol 1.25 mg	Yes
22	D	M	Hyperglycaemia	1	Glibenclamide 5 mg	Yes
23	P	L	HTN	1	Bisoprolol 5 mg	No
24	D	L	Hyperglycaemia	1	Insulin rapid	Yes
25	D	L	Cough	1	Lisinopril 10 mg	Yes
26	P	L	HTN	1	Bisoprolol 7.5 mg<br>Furosemide 80 mg	No
27	D	L	Epigastric pain	1	Aspirin 75 mg	Yes
28	D<br>D	M<br>M	Anaemia no iron intake<br>Hyperglycaemia	2	Iron<br>Glibenclamide 5 mg	Yes<br>Yes
29	D	L	Hyperglycaemia	1	Novomix 30:14 units	Yes
30	D	L	Cough	1	Simvastatin 40 mg	No
31	D	M	Tachycardia	1	Thyroid hormone	Yes
32	P	L	Tachycardia	1	Levothyroxin 100 mcg	Yes
33	D<br>D	No harm<br>No harm	Hyperlipidaemia<br>anaemia	2	Simvastatin 20 mg<br>Missing medicine	No<br>No
34	D<br>P	L<br>S	Hyperglycaemia<br>Epigastric pain	2	Insulatard<br>Clopidogrel 75 mg	Yes<br>No
35	D<br>D	L<br>M	Infection<br>hyperglycaemia	2	Augmentin 500 mg<br>Metformin 500 mg	Yes<br>No
36	D<br>D	L<br>S	Hyperglycaemia<br>Gastroenteritis	2	Insulin mixtard<br>Aspirin 75 mg	No<br>Yes
37	D	No harm	Hyperglycaemia	1	Missing medicine	No
38	D	No harm	Hyperglycaemia	1	Missing medicine	No
39	D	No harm	Hyperglycaemia	1	Insulatard mix<br>Metformin 50 mg	No
40	D<br>D	L<br>M	Hyperglycaemia<br>Uncontrolled blood pressure	2	Insulin mix<br>Ramipril 1.25 mg	No<br>Yes
41	D	L	Hyperglycaemia	1	Missing medicine	Yes
42	D	No harm	Hyperglycaemia	1	Missing medicine	No

Table S2. *Cont.*

Patient Number	Type	Severity	MRP	*N*	Medicine	Cause of Admission
43	D	No harm	Hyperglycaemia	1	Insulin NPH<br>Insulin regular	No
44	D	No harm	Hyperglycaemia	1	Missing medicine	No
45	D	L	Hyperglycaemia	1	Missing medicine	Yes
46	D<br>P	L harm<br>No harm	Hyperglycaemia<br>Elevated triglycerides	2	Missing medicine	No<br>No
47	D<br>P	No harm<br>No harm	Hyperglycaemia<br>Abdominal pain	2	Missing medicine<br>Aspirin 75 mg	No
48	D	No harm	Hyperglycaemia	1	Glibenclamide 5 mg	No
49	D	No harm	Hyperglycaemia	1	Insulin regular	No
50	D	No harm	Hyperglycaemia	1	Insulin mix	No
51	D	L	Hyperglycaemia	1	Missing medicine	Yes
52	P	No harm	Hyperglycaemia	1	Missing medicine	No
53	P	No harm	HTN	1	Bisoprolol 3.75 mg<br>Furosemide 40 mg	No
54	D<br>D	No harm<br>No harm	Hyperglycaemia<br>Elevated triglycerides	2	Missing medicines<br>Simvastatin 40 mg	Yes<br>No
55	P	No harm	Hyperglycaemia	1	Insulin mix	No
56	D	L	Hyperglycaemia	1	Missing medicine	Yes
57	P	No harm	Productive cough/infection	1	Azithromycin 500 mg	No
58	D	No harm	Hypertension	1	Metoprolol 12.5 mg	No
59	D	No harm	Anaemia	1	Iron<br>Folic acid 5 mg	No
60	D	L	Hyperglycaemia	1	Missing medicine	Yes
61	D<br>D	No harm<br>S	Hyperglycaemia<br>Theophylline toxicity	2	Gliclazide 160 mg<br>Theophylline 150 mg	No<br>Yes
62	D	M	Uncontrolled HTN	1	Amlodipine 10 mg<br>Furosemide 20 mg<br>Isosorbide dinitrate 20 mg<br>Metoprolol 25 mg<br>Perindopril 5 mg	Yes
63	D	M	Digitalis toxicity	1	Digoxin 125 mcg	Yes
64	D	No harm	Hyperglycaemia	1	Insulin mix	No

Table S2. *Cont*.

Patient Number	Type	Severity	MRP	*N*	Medicine	Cause of Admission
65	P<br>P	No harm<br>No harm	Hyperglycaemia<br>Palpitations	2	Insulin rapid<br>Metoprolol 25 mg	No
66	D	No harm	Hyperglycaemia	1	Insulin mix	No
67	D	L	Hyperglycaemia	1	Missing medicine	No
68	D	L	Hyperglycaemia	1	Insulin	Yes
69	D	L	Hyperglycaemia	1	Insulin mix	Yes
70	P	No harm	High cholesterol	1	Simvastatin 10 mg	No
71	P	No harm	Vomiting	1	Aspirin 324 mg	No
72	P	no harm	Cough	1	Simvastatin 10 mg	No
73	D<br>D	L<br>M	Hyperglycaemia<br>Bradycardia	2	Insulin<br>Tamsulosin 0.4 mg	Yes<br>Yes
74	D	L	HTN	1	Nifedipine 30 mg<br>Valsartan 160 mg	Yes
75	P	L	Hypoglycaemia	1	Missing medicine	No
76	D	L	Hyperglycaemia	1	Missing medicine	No
77	P	L	HTN	1	Missing medicine	Yes
78	P	No harm	Orthopnoea	1	Aspirin 81 mg<br>Furosemide 40 mg	NA
79	D	L	Hyperglycaemia	1	Insulin rapid	Yes
80	D	S	Gastric bleeding	1	Aspirin 75 mg	Yes
81	D	L	HTN	1	Lisinopril 10 mg	Yes
82	D	M	Abdominal pain	1	Aspirin 75 mg<br>Clopidogrel 75 mg	Yes

© 2016 by the authors; licensee MDPI, Basel, Switzerland. This article is an open access article distributed under the terms and conditions of the Creative Commons by Attribution (CC-BY) license (http://creativecommons.org/licenses/by/4.0/).
